# Residual metric learning with class-specific consistency for multiclass classification

**DOI:** 10.1371/journal.pone.0345369

**Published:** 2026-03-25

**Authors:** Kai Hu, Jiajun Ma

**Affiliations:** School of Computer Science and Engineering, Xi’an Technological University, Xi’an, Shaanxi, China; Northwestern Polytechnical University, CHINA

## Abstract

Least squares regression (LSR) has been widely used in pattern recognition due to its concise form and ease of solution. However, inadequate exploration of inter-class margin and intra-class similarity limits its discriminative ability. To this end, we present a novel method called residual metric learning with class-specific consistency for multiclass classification (RMLCC). Specifically, RMLCC jointly learns a projection matrix and a metric matrix for the regression residuals in a compact framework. This joint learning mechanism makes the inter-class margin of the projected instances as large as possible in the learned metric space, prompting the instances of different classes to be separated. To further improve the generalization, the class-specific consistency constraint that stimulate intra-class similarity is cleverly embedded into the joint learning framework. To solve the proposed model, we propose an alternative optimization algorithm which guarantees weak convergence. With the interactive optimization of the projection matrix and metric matrix, RMLCC can fully exploit the structure and supervised information of the data and thus has the potential to outperform other methods. Extensive experiments on several benchmark datasets demonstrate the validity of the proposed method.

## Introduction

Least squares regression (LSR) [1] has been widely used in many applications, such as image recognition [2] and discriminative learning [3], due to its concise formulation and efficient solution. By embedding some given prior information into the objective function, LSR can also be tailored for different tasks such as regression [1] or classification [4]. Here, we focus on the task of multiclass classification, where one instance is assigned to one of a number of discrete classes. Broadly speaking, a good classifier should perform well in terms of discrimination and generalization. The conventional LSR-based classification model aims to learn a mapping function that projects the input instances into the binary label space by solving a mean square error minimization problem. This operation not only degrades the discrimination performance but also easily leads to overfitting [1,5], as it encourages a constant Euclidean distance between the regression responses of any two instances from different classes and does not consider the intra-class similarity.

To enhance the discrimination, label relaxation techniques pursuing large inter-class margin have been successively explored. Xiang et al. [2] proposed a discriminative LSR (DLSR) model, which enlarges the distance between regression targets of different classes using the *ε*-dragging technique. Wang et al. [6] presented a margin scalable discriminative LSR (MSDLSR) method by introducing a sparsity constraint on the dragging values. Zhang et al. [7] constructed a retargeted least squares regression (ReLSR) model, which directly learns the regression targets with large inter-class margin. To improve the generalization, various sparse or low rank regularization terms are introduced into the objective function to maintain the intra-class similarity or structural consistency. In [8], a discriminative LSR method based on inter-class sparsity (ICS_DLSR) was proposed. ICS_DLSR introduces an inter-class sparsity constraint to reduce the intra-class margin while increasing the inter-class margin, and introduces the error factors to improve the discriminability of the model. A group low-rank representation-based discriminative linear regression (GLRRDLR) model was presented by Zhan et al. [9], which imposed a class-wise low-rank constraint on the latent features.

In addition, several subspace learning methods have also been developed to extract the structural information and improve the generalization. Fang et al. [10] presented a robust latent subspace learning (RLSL) model through the combination of latent representation with linear regression. Zhang et al. [11] proposed a pairwise relations oriented discriminative regression (PRDR) model, in which the pairwise relations of the label and the instances are transferred to the latent subspace by means of cosine similarity and manifold regularization. These methods explore the manifold structure of the instance in the latent subspace under the guidance of a pre-computed similarity graph. However, those pre-computed graphs often employ features from original data and cannot be adaptively learned during the training to better express the structures of instances. To make matters worse, constructing a graph Laplacian with a simple weight function is not discriminative enough, but using the complex weight function increases the complexity exponentially. Zadeh et al. [12] described the distance structure between instances from the perspective of metric learning, and proposed a geometric mean metric learning (GMML) model. Unlike the manifold learning based methods, GMML minimizes an unconstrained strictly (geodesically) convex optimization problem, allowing for a closed-form solution that yields smaller distances for similar instances and larger distances for dissimilar instances.

Although the above methods try to pursue the large inter-class margin and intra-class consistency, they still cannot accommodate both interclass separability and intraclass similarity because the transformation matrix learning and structure exploring are independent. How to effectively exploit the structural information and label relations to learn the discriminative representation remains to be explored. In this paper, we propose a novel model, referred to as residual metric learning with class-specific consistency for multiclass classification (RMLCC). In particular, we jointly learn a projection matrix and a metric matrix for the regression residuals, which allows the inter-class margin of the projected instances to be as large as possible in the learned metric space. Furthermore, a class-specific consistency constraint is cleverly embedded in the joint learning framework to ensure the intra-class similarity. In this way, the discriminant projection matrix and the residual metric matrix are mutually reinforcing. This allows the model to account for both the inter-class separability and intra-class similarity. The key contributions of the RMLCC are outlined as follows.

- A joint learning framework is proposed to learn a projection matrix and a metric matrix for its residuals. This operation actually constructs an adaptive loss function based on the learnt metric matrix, aiming to separate instances of different classes and gather together instances of the same class as much as possible.
- A class-specific consistency regularisation is introduced into the joint learning framework to ensure that instances of the same class remain structurally consistent after projection, fully exploiting the similarity between instances of the same class and thus alleviates the overfitting problem.
- The solution to the objective function is studied and the corresponding algorithm is developed. Experiments are performed on six benchmark datasets and compared with seven comparison methods to evaluate the performance of the proposed method.

The rest of this paper is organised as follows. Section Related work provides a brief overview of the relevant methods. Section The proposed method presents our proposed method in detail. The experimental results and analysis are reported in Section Experiment. The conclusion is finally given in Section Conclusion.

## Related work

We first introduce the notations used in this paper, and then provide a review of some related works. For matrix $𝐀\in {ℜ}^{m\times n}$, **A**_*i*,*j*_ denotes the (*i*,*j*)-th element of **A**, **A**_*i*,:_ and **A**_:,*j*_ denote the *i*-th row vector and *j*-th column vector, respectively. $\|𝐀{\|}_{1,2}=\sum_{j}\sqrt{\sum_{i}|{𝐀}_{i,j}{\|}^{2}}$ denotes the *l*_1,2_-norm. The square Frobenius norm is defined as $\left\|𝐀{\|}_{F}^{2}=tr({𝐀}^{⊤}𝐀\right)$, where tr(**A**) is the trace of **A**. For a symmetric positive definite (PSD) matrix **M**, ${𝐌}^{-1}$ is the inverse of **M**, $\left\|𝐀{\|}_{𝐌}^{2}=tr({𝐀}^{⊤}𝐌𝐀\right)$.

Denote a set of *n* instances with *d* features by $𝐗=[x_{1},\ldots ,x_{n}{]}^{⊤}\in {ℜ}^{n\times d}$. The binary label indicator matrix is defined as $𝐘=[y_{1},y_{2},\ldots ,y_{n}{]}^{⊤}\in {ℜ}^{n\times c}$, where $y_{i}=[0,\ldots ,1,\ldots ,0{]}^{⊤}$ and the position of 1 indicates the belonging class of $x_{i}$, *c* is the number of classes. Record the instances from *j*th class and their labels as **X**_*j*_ and **Y**_*j*_, and the instances excluding class *j* and their labels as ${𝐗}_{\bar{j}}$ and ${𝐘}_{\bar{j}}$. *n*_*j*_ denotes the number of instances from *j*th class, $n_{\bar{j}}=n-n_{j}$.

### LSR and DLSR

For the multiclass classification task, LSR seeks an optimal projection matrix by solving the following problem:


$$\min_{𝐖}\|𝐗𝐖-𝐘{\|}_{F}^{2}+\lambda \|𝐖{\|}_{F}^{2},$$ (1)

where the bias is absorbed into **X** as an additional dimension with all elements equal to 1, **W** is the projection matrix to be learned, and *λ* is a regularization parameter. Eq.(1) is strictly convex and admits a closed-form solution. Intuitively, it is usually hoped that instances of different classes will be as far apart from each other as possible after projection, while instances of the same class will be closer together after projection, which is beneficial for subsequent classification. However, Eq.(1) encourages a constant distance of $\sqrt{2}$ between the regression responses of any two instances in different classes, and does not take into account the similarity between instances belonging to the same class. Therefore, the discrimination of LSR in multiclass classification still needs to be improved.

To further enhance the discriminative capability, DLSR relaxes the binary label indicator matrix and drags the regression targets of different classes along the opposite directions using a “*ε*-dragging” technique. The particular model is


$$\min_{\text{W},\text{E}}\|\text{XW}-\text{Y}-\text{B}⊙\text{E}{\|}_{F}^{2}+\lambda \|\text{W}{\|}_{F}^{2},\;\text{s.t.}\text{E}\geq 0$$ (2)

where ⊙ denotes the Hadamard product operator for matrices, $\text{E}=({\text{E}}_{i,j})\in {\mathbb{R}}^{n\times c}$ is a nonnegative dragging matrix and calculated by E=max(B⊙(XW−Y),0). The matrix **B** is defined as follows:


$$\begin{array}{c} {\text{B}}_{i,j}=\left\{ \begin{array}{cc} +1, & {𝐘}_{i,j}=1,\\ -1, & {𝐘}_{i,j}=0. \end{array}\right.\; \end{array}$$ (3)

The objective Eq.(2) is jointly convex with respect to **W** and **E**, and can be solved through alternating optimization. Although the “*ε*-dragging” relaxation can enhance the inter-class separability, it can also cause instances from the same class to be uncorrelated after projection, as it does not consider the intra-class compactness.

### GMML

Unlike the above methods that use the binary labels directly as supervised information, Zadeh et al. [12] proposed a geometric mean metric learning method to measure the structural relationships between instances using the side information of the paired instances. The formula is


$$\min_{𝐌≻0}\sum_{(x_{i},x_{j})\in 𝒮}d_{𝐌}^{2}(x_{i},x_{j})+\sum_{(x_{i},x_{j})\in 𝒟}d_{{𝐌}^{-1}}^{2}(x_{i},x_{j})+\mu D_{sld}(𝐌,{𝐌}_{0}),$$ (4)

Here, **M** is a symmetric positive definite (SPD) matrix to be learned, **M**_0_ is a prior SPD matrix about **M**. 𝒮 and 𝒟 record the similar and dissimilar instance sets, respectively. $d_{𝐌}^{2}(x_{i},x_{j})=(x_{i}-x_{j}{)}^{⊤}𝐌(x_{i}-x_{j})$ denotes the square Mahalanobis distance between $x_{i}$ and $x_{j}$, *μ* is a regularization parameter. $D_{sld}(𝐌,{𝐌}_{0})$ is the symmetrized LogDet divergence. Eq.(4) is a strictly (geodesically) convex optimization problem with the closed-form solution


$$𝐌=(𝐆+\mu 𝐈{)}^{-1}{♯}_{\alpha }(𝐏+\mu 𝐈),$$ (5)

where $𝐆=\sum_{(x_{i},x_{j})\in 𝒮}(x_{i}-x_{j})(x_{i}-x_{j}{)}^{⊤}$, $𝐏=\sum_{(x_{i},x_{j})\in 𝒟}(x_{i}-x_{j})(x_{i}-x_{j}{)}^{⊤}$, ${♯}_{\alpha }$ is the geometric mean operator [12], and α∈(0,1) is a weighting parameter.

## The proposed method

**Fig 1:**
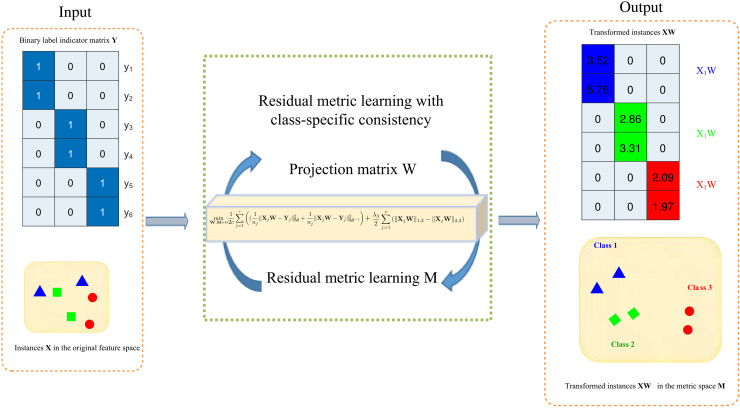
Overview of the structure of RMLCC. We learn the projection matrix **W** and metric matrix **M** simultaneously, so that the transformed instances of different classes can be easily separated in the learned metric space.

Fig 1 illustrates the framework of our RMLCC. Different from DLSR and GMML, our RMLCC jointly learns the projection matrix and metric matrix for the regression residuals in a unified framework. RMLCC jointly learns a transformation matrix and a metric matrix, such that the metric matrix yields smaller distances for the regression residuals of the same class and larger distances for different classes. To further improve the discriminative performance, a class-specific consistency constraint is imposed on the transformed instances of each class.

### Target-margin dragging

To enhance discrimination, we enlarge the inter-class targets’ margins while reducing those from the same class. Considering that GMML [12] has achieved great success in metric learning and fit our goal well, we adopt a similar way to drag the regression margins in our model. Certainly, other metric learning techniques can replace GMML, but that is beyond our focus.

For convenience, we first reformulate (1) in an equivalent form as


$$\;\;\min_{𝐖}\;\;\frac{1}{c}\;\sum_{j=1}^{c}\;(\;\|{𝐗}_{j}𝐖-{𝐘}_{j}{\|}_{F}^{2}\;+\;\|{𝐗}_{\bar{j}}𝐖-{𝐘}_{\bar{j}}{\|}_{F}^{2}\;)\;+\;\;\lambda \|𝐖\;{\|}_{F}^{2}\;\;$$ (6)

To conduct target-margin dragging, we learn a metric matrix **M** such that the regression errors for the same class are as small as possible under its metric, while the errors for the remaining classes are as large as possible under its metric. Then we have


$$\min_{𝐖,𝐌≻0}\frac{1}{c}\sum_{j=1}^{c}\left(\frac{1}{n_{j}}\;\|{𝐗}_{j}𝐖-{𝐘}_{j}{\|}_{𝐌}^{2}\;+\;\frac{1}{n_{\bar{j}}}\|{𝐗}_{\bar{j}}𝐖-{𝐘}_{\bar{j}}{\|}_{{𝐌}^{-1}}^{2}\right)+\lambda_{1}D_{sld}(𝐌,{𝐌}_{0})\;\;+\;\lambda_{2}\|𝐖\;{\|}_{F}^{2},\;$$ (7)

where $\|\cdot {\|}_{𝐌}^{2}$ and $\|\cdot {\|}_{{𝐌}^{-1}}^{2}$ denote the square Mahalanobis distance under the metric of **M** and ${𝐌}^{-1}$, respectively. To alleviate the problem of class imbalance, proportional coefficients $\frac{1}{n_{j}}$ and $\frac{1}{n_{\bar{j}}}$ were incorporated into the first and second terms in (7), respectively.

It is worth noting that we perform metric learning for the residuals rather than the instances themselves, which enables the discriminant projection and metric matrix for the instances to be synchronized and mutually reinforcing. By embedding metric **M** between errors of the same class and its inverse ${𝐌}^{-1}$ between errors of different classes, the regressor’s discriminability can be enhanced.

### Class-specific consistency

When binary labels are used as the regression targets, the desired output of the *j*th class instances **X**_*j*_ are


$$\begin{array}{c} {𝐗}_{j}𝐖={\left[\begin{array}{ccccc} 0 & \cdots & 1 & \cdots & 0\\ 0 & \cdots & 1 & \cdots & 0\\ \vdots & \ddots & \vdots & \ddots & \vdots \\ 0 & \cdots & 1 & \cdots & 0 \end{array}\right]}_{n_{j}\times c},\; \end{array}$$ (8)

where 1s are on the *j*th column. The index of the non-zero column indicates the identity of the class of the collection of instances **X**_*j*_.

It is clear from (8) that the output matrix of the instances from class *j* has a consistently sparse structure, i.e., only the *j*th column is non-zero. The regression output **X**_*j*_**W** of the instances **X**_*j*_ have the same index of non-zero entries, which promotes the consistency structure for the same class. To this end, we first introduce the class-specific consistency regularization with the following proposition.

**Proposition 1** *For any non-zero matrix* ***A****, $\|𝐀{\|}_{1,2}-\|𝐀{\|}_{2,2}\geq 0$ if and only if* ***A*** *has one single nonzero column, $\|𝐀{\|}_{1,2}-\|𝐀{\|}_{2,2}=0$.*

*Proof.* Please refer to the Appendix S1 Appendix for the detailed proof of Proposition 1. □

As stated in Proposition 1, the class-specific consistency regularization satisfies our search for intra-class similarity, so we use it for the transformed instances, i.e.,


$$min\sum_{j=1}^{c}(\|{𝐗}_{j}𝐖{\|}_{1,2}-\|{𝐗}_{j}𝐖{\|}_{2,2}).$$ (9)

### Objective function

Combining the target-margin dragging in (7) with the class-specific consistency regularization in (9), the residual metric learning with class-specific consistency for multiclass classification is formulated as


$$\begin{array}{cc} & \;\;\min_{𝐖,𝐌≻0}\frac{1}{2c}\sum_{j=1}^{c}\left(\;\frac{1}{n_{j}}\|{𝐗}_{j}𝐖\;-\;{𝐘}_{j}{\|}_{𝐌}^{2}+\frac{1}{n_{\bar{j}}}\|{𝐗}_{\bar{j}}𝐖\;-\;{𝐘}_{\bar{j}}{\|}_{{𝐌}^{-1}}^{2}\right)\\ & \;\;\;+\frac{\lambda_{1}}{2}\;D_{sld}(𝐌,{𝐌}_{0})\;+\;\frac{\lambda_{2}}{2}\|𝐖\;{\|}_{F}^{2}+\lambda_{3}\sum_{j=1}^{c}\left(\|{𝐗}_{j}𝐖{\|}_{1,2}\;-\;\|{𝐗}_{j}𝐖{\|}_{2,2}\;\right)\; \end{array}$$ (10)

Model (10) enjoys the following three valuable properties:

- The first term $\frac{1}{n_{j}}\|{𝐗}_{j}𝐖-{𝐘}_{j}{\|}_{𝐌}^{2}$ is the square regression residuals of *j*-th class under the **M** metric, which increases monotonically with respect to **M**. Thus, minimizing this term results in a small distance for the same class.
- The second item $\frac{1}{n_{\bar{j}}}\|{𝐗}_{\bar{j}}𝐖-{𝐓}_{\bar{j}}{\|}_{{𝐌}^{-1}}^{2}$ is the regression residuals of classes excluded in *j*-th class, which decreases monotonically with respect to **M**. Thus, minimizing this term results in a large distance for the different classes.
- The last terms $\|{𝐗}_{j}𝐖{\|}_{1,2}-\|{𝐗}_{j}𝐖{\|}_{2,2}$ denote the class-specific consistency regularization term, which ensures the structural consistency for the transformed instances of the same class.

Since **M** is a SPD matrix, (10) actually finds a more discriminative transformation $𝐖\tilde{𝐌}$ for **X**, where $\tilde{𝐌}$ satisfies $𝐌=\tilde{𝐌}{\tilde{𝐌}}^{⊤}$. Our RMLCC is not a simple combination of LSR and GMML, which actually employees the adaptive Mahalanobis distance $d_{𝐌}^{2}(\cdot )$ as the loss function rather than the Frobenius norm $\|\cdot {\|}_{F}^{2}$ used in LSR.

### Optimization

Using matrix trace operation, (10) can be simplified and reformulated as


$$\begin{array}{cc} & \;\;\min_{𝐖,𝐌≻0}\frac{1}{2}tr\left(𝐌(𝐗𝐖-𝐘{)}^{⊤}𝐋(𝐗𝐖-𝐘)\right)+\frac{1}{2}tr\left({𝐌}^{-1}(𝐗𝐖-𝐘{)}^{⊤}𝐃(𝐗𝐖-𝐘)\right)\\ & \;\;\;+\frac{\lambda_{1}}{2}\;D_{sld}(𝐌,{𝐌}_{0})+\frac{\lambda_{2}}{2}\|𝐖{\|}_{F}^{2}+\lambda_{3}\sum_{j=1}^{c}\left(\|{𝐗}_{j}𝐖{\|}_{1,2}-\|{𝐗}_{j}𝐖{\|}_{2,2}\right)\; \end{array}$$ (11)

where **L** = diag$\left(\frac{1}{cn_{l(x_{1})}},\ldots ,\frac{1}{cn_{l(x_{n})}}\right)$, and **D** = diag$\left(\sum_{j\neq l(x_{1})}^{c}\frac{1}{cn_{\bar{j}}},\ldots ,\sum_{j\neq l(x_{n})}^{c}\frac{1}{cn_{\bar{j}}}\right)$. Here, *c* denotes the number of classes, $l(x_{j})$ is the label of $x_{j}$, $n_{l(x_{j})}$ recorded the number of instances associated with label $l(x_{j})$, and $n_{\bar{j}}$ recorded the number of instances not associated with label *j*.

The optimization problem (11) is nonconvex and involves two variables. Here, an iterative algorithm based on ADMM [13–15] framework is employed to solve problem (11). First, two auxiliary variables **U** and **F** are introduced to make the optimization problem (11) separable as follows:


$$\begin{array}{cc} & \;\;\min_{𝐖,𝐌,𝐔,𝐅}\frac{1}{2}tr\left(𝐌(𝐗𝐖-𝐘{)}^{⊤}𝐋(𝐗𝐖-𝐘)\right)+\frac{1}{2}tr\left({𝐌}^{-1}(𝐗𝐔-𝐘{)}^{⊤}𝐃(𝐗𝐔-𝐘)\right)\\ & \;\;\;+\frac{\lambda_{1}}{2}\;D_{sld}(𝐌,{𝐌}_{0})+\frac{\lambda_{2}}{2}\|𝐔\;{\|}_{F}^{2}+\lambda_{3}\sum_{j=1}^{c}\left(\|{𝐅}_{j}{\|}_{1,2}-\|{𝐅}_{j}{\|}_{2,2}\right)\\ & \;\;\text{s.t.}𝐌≻0,𝐖=𝐔,𝐗𝐖=𝐅\; \end{array}$$ (12)

Then, we obtain the following augmented Lagrangian function of Eq.(12)


$$\begin{array}{cc} {ℒ}_{\mu ,\sigma }= & \;\;\frac{1}{2}tr\left(𝐌(𝐗𝐖-𝐘{)}^{⊤}𝐋(𝐗𝐖-𝐘)\right)+\frac{1}{2}tr\left({𝐌}^{-1}(𝐗𝐔-𝐘{)}^{⊤}𝐃(𝐗𝐔-𝐘)\right)\\ & \;\;\;+\frac{\lambda_{1}}{2}\;D_{sld}(𝐌,{𝐌}_{0})+\frac{\lambda_{2}}{2}\|𝐔\;{\|}_{F}^{2}+\lambda_{3}\sum_{j=1}^{c}\left(\|{𝐅}_{j}{\|}_{1,2}-\|{𝐅}_{j}{\|}_{2,2}\right)\\ & \;\;\;+\frac{\mu }{2}\|𝐖-𝐔+\frac{𝐏}{\mu }{\|}_{F}^{2}+\frac{\sigma }{2}\|𝐗𝐖-𝐅+\frac{𝐙}{\sigma }{\|}_{F}^{2},\; \end{array}$$ (13)

where $𝐅=[F_{1};\ldots ;F_{c}]=[{𝐗}_{1}𝐖;\ldots ;{𝐗}_{c}𝐖]$, **P** and **Z** are Lagrange multipliers, μ>0 and σ>0 are penalty factors. Next, the variables will be updated alternately.

**Step 1.** Fixing other variables, **W** is updated by minimizing the following problem

$$\begin{array}{cc} 𝒥(𝐖) & \;\;=\frac{1}{2}tr\;\left(\;𝐌(𝐗𝐖-𝐘{)}^{⊤}𝐋(𝐗𝐖-𝐘)\;\right)\\ & \;\;\;+\frac{\mu }{2}\|𝐖-𝐔+\frac{𝐏}{\mu }{\|}_{F}^{2}+\frac{\sigma }{2}\|𝐗𝐖-𝐅+\frac{𝐙}{\sigma }{\|}_{F}^{2}\; \end{array}$$ (14)

By setting the derivative (∂𝒥(𝐖)/∂𝐖)=0, we obtain


$${𝐗}^{⊤}𝐋𝐗𝐖+(\mu 𝐈+\sigma {𝐗}^{⊤}𝐗)𝐖{𝐌}^{-1}=𝐂,$$ (15)

where $𝐂={𝐗}^{⊤}𝐋𝐘+(\mu 𝐔-𝐏+\sigma {𝐗}^{⊤}𝐅-{𝐗}^{⊤}𝐙)M^{-1}$, **W** is updated by solving a Sylvester equation [16].

**Step 2.** Fixing other variables, **U** is updated by solving the following problem:

$$𝒥(𝐔)=\frac{1}{2}tr\left({𝐌}^{-1}(𝐗𝐔-𝐘{)}^{⊤}𝐃(𝐗𝐔-𝐘)\right)+\frac{\lambda_{1}}{2}\|𝐔{\|}_{F}^{2}+\frac{\mu }{2}\|𝐖-𝐔+\frac{𝐏}{\mu }{\|}_{F}^{2}.$$ (16)

By setting the derivative (∂𝒥(𝐔)/∂𝐔)=0, we obtain


$${𝐗}^{⊤}𝐃𝐗𝐔+(\lambda_{1}+\mu )𝐔𝐌=𝐄,$$ (17)

where $𝐄={𝐗}^{⊤}𝐃𝐘+(\mu 𝐖+𝐏)M$, **U** is updated by solving a Sylvester equation.

**Step 3.** Fixing other variables, **F** is updated by solving the following problem:

$$𝒥(𝐅)=\lambda_{3}\sum_{j=1}^{c}\left(\|{𝐅}_{j}{\|}_{1,2}-\|{𝐅}_{j}{\|}_{2,2}\right)+\frac{\sigma }{2}\|𝐗𝐖-𝐅+\frac{𝐙}{\sigma }{\|}_{F}^{2},$$ (18)

Obviously, the problem can be solved for each **F**_*j*_ independently, where **F**_*j*_ is the *j*th subset of **F**. If we define $𝐇=𝐗𝐖+\frac{𝐙}{\sigma }$, then the optimization problem (18) is equivalent to the following problems:


$$\begin{array}{cc} & \;\;𝒥({𝐅}_{j})=\lambda_{3}\left(\|{𝐅}_{j}{\|}_{1,2}-\|{𝐅}_{j}{\|}_{2,2}\right)+\frac{\sigma }{2}\|{𝐇}_{j}-{𝐅}_{j}{\|}_{F}^{2},\\ & \;\;\;forj=1,2,\ldots ,c\; \end{array}$$ (19)

where **H**_*j*_ is the *j*th subset of **H**, corresponding to the instances from the *j*th class. Let $f_{k}$ and $h_{k}$ the *k*th column of **F**_*j*_ and **H**_*j*_ respectively, then we have $\|{𝐅}_{j}{\|}_{1,2}=\sum_{k=1}^{c}\|f_{k}{\|}_{2}$ and $\|{𝐅}_{j}{\|}_{2,2}=\frac{\sum_{k=1}^{c}\|f_{k}{\|}_{2}^{2}}{(\sum_{k=1}^{c}\|f_{k}{\|}_{2}^{2}{)}^{1/2}}$. The objective of problem (19) can be reformulated as


$$\begin{array}{cc} \;𝒥({𝐅}_{j}) & \;\;=\lambda_{3}\sum_{k=1}^{c}(\|f_{k}{\|}_{2}\;-\;\frac{\|f_{k}{\|}_{2}^{2}}{(\sum_{k=1}^{c}\|f_{k}{\|}_{2}^{2}{)}^{1/2}})\;+\;\frac{\sigma }{2}\sum_{k=1}^{c}\|h_{k}\;-\;f_{k}{\|}_{2}^{2}\\ & \;\;=\lambda_{3}\sum_{k=1}^{c}(\frac{1}{\|f_{k}{\|}_{2}}\;-\;\frac{1}{(\sum_{k=1}^{c}\|f_{k}{\|}_{2}^{2}{)}^{1/2}})\|f_{k}{\|}_{2}^{2}+\frac{\sigma }{2}\sum_{k=1}^{c}\|h_{k}\;-\;f_{k}{\|}_{2}^{2}\;\;\; \end{array}$$ (20)

To simplify the problem (20), we linearize it as follows:


$$\;\tilde{𝒥}({𝐅}_{j})\;=\;\lambda_{3}\sum_{k=1}^{c}(\frac{1}{\|f_{k}^{t}{\|}_{2}}\;-\;\frac{1}{(\sum_{k=1}^{c}\|f_{k}^{t}{\|}_{2}^{2}{)}^{1/2}})\|f_{k}{\|}_{2}^{2}+\frac{\sigma^{t}}{2}\sum_{k=1}^{c}\|h_{k}\;-\;f_{k}{\|}_{2}^{2}\;\;$$ (21)

where $f_{k}^{t}$ is the result of variable $f_{k}$ at the *t*-th iteration. By setting the derivative $(\partial \tilde{𝒥}({𝐅}_{j})/\partial f_{k})=0$, we obtain


$$2\lambda_{3}(\frac{1}{\|f_{k}^{t}{\|}_{2}}\;-\;\frac{1}{(\sum_{k=1}^{c}\|f_{k}^{t}{\|}_{2}^{2}{)}^{1/2}})f_{k}-\;\sigma^{t}(h_{k}\;-\;f_{k})=0\;\;$$ (22)

Then,


$$\begin{array}{cc} f_{k}^{t+1} & \;\;=\frac{\sigma^{t}h_{k}}{\sigma^{t}+2\lambda_{3}(\frac{1}{\|f_{k}^{t}{\|}_{2}}\;-\;\frac{1}{(\sum_{k=1}^{c}\|f_{k}^{t}{\|}_{2}^{2}{)}^{1/2}})}\\ & \;\;=\frac{\sigma^{t}h_{k}}{(\sigma^{t}-\;\frac{2\lambda_{3}}{(\sum_{k=1}^{c}\|f_{k}^{t}{\|}_{2}^{2}{)}^{1/2}})+\frac{2\lambda_{3}}{\|f_{k}^{t}{\|}_{2}}}\; \end{array}$$ (23)

Define $\beta_{1}=\sigma^{t}-\;\frac{2\lambda_{3}}{(\sum_{k=1}^{c}\|f_{k}^{t}{\|}_{2}^{2}{)}^{1/2}}$, $\beta_{2}=\frac{2\lambda_{3}}{\|f_{k}^{t}{\|}_{2}}$, finally


$$f_{k}^{t+1}=\frac{\sigma^{t}}{\beta_{1}+\beta_{2}}h_{k},$$ (24)

that is ${𝐅}_{j}^{\left(t+1\right)}=[f_{1}^{t+1},\ldots ,f_{k}^{t+1},f_{c}^{t+1}]$.

**Step 4.** Fixing other variables, **M** is updated by solving the following problem:

$$\begin{array}{cc} 𝒥(𝐌) & \;\;=\min_{𝐌}\frac{1}{2}tr\left(𝐌(𝐗𝐖-𝐘{)}^{⊤}𝐋(𝐗𝐖-𝐘)\right)\\ & \;\;\;+\frac{1}{2}tr\left({𝐌}^{-1}(𝐗𝐔-𝐘{)}^{⊤}𝐃(𝐗𝐔-𝐘)\right)\\ & \;\;\;+\frac{\lambda_{1}}{2}\;D_{sld}(𝐌,{𝐌}_{0})\; \end{array}$$ (25)

Eq. (25) enjoys a closed-form solution


$$𝐌=(𝐆+\lambda_{1}{𝐌}_{0}^{-1}{)}^{-1}{♯}_{\alpha }(𝐒+\lambda_{1}{𝐌}_{0}),$$ (26)

where ${♯}_{\alpha }$ defined as $𝐀{♯}_{\alpha }𝐁={𝐀}^{1/2}({𝐀}^{-1/2}𝐁{𝐀}^{-1/2}{)}^{\alpha }{𝐀}^{1/2}$, α∈(0,1) is a weighting parameter, and


$$\begin{array}{cc} & \;\;𝐆=\frac{1}{2}(𝐗𝐖-𝐘{)}^{⊤}𝐋(𝐗𝐖-𝐘),\\ & \;\;𝐒=\frac{1}{2}(𝐗𝐔-𝐘{)}^{⊤}𝐃(𝐗𝐔-𝐘).\; \end{array}$$

**Step 5.** The Lagrange multiplier **P**, **Z**, and penalty factors *μ*, *σ* are updated as:

$$\begin{array}{cc} 𝐏 & \;\;=𝐏+\mu (𝐖-𝐔),\mu =min(\rho \mu ,\mu_{max})\\ 𝐙 & \;\;=𝐙+\sigma (𝐗𝐖-𝐅),\sigma =min(\rho \sigma ,\sigma_{max})\; \end{array}$$ (27)

In steps 1–2, ${𝐗}^{⊤}𝐋𝐗$ and ${𝐗}^{⊤}𝐃𝐗$ are fixed in iterations, we can implement the Schur decomposition for them and store the results in advance to speedup. The pseudocode implementation of RMLCC is shown in Algorithm 1.

**Algorithm 1 Algorithm for Solving RMLCC.**

**Input** Training instance matrix **X**, label matrix **Y**, parameters $\lambda_{1}$, $\lambda_{2}$, $\lambda_{3}$.

**Output** Transform matrix **W** and metric matrix **M**.

1:Initialization: **W** with random values, **M** with identity matrix, **U** = **W**, **F** = **XW**, **Z** = **0**, **P** = **0**, $\mu_{max}=10^{5}$, $\sigma_{max}=10^{5}$, ρ=1.1

2:Perform Schur decomposition for ${𝐗}^{⊤}𝐋𝐗$ and ${𝐗}^{⊤}𝐃𝐗$, ${𝐗}^{⊤}𝐋𝐗={𝐐}_{1}\Lambda_{1}{𝐐}_{1}^{⊤}$, ${𝐗}^{⊤}𝐃𝐗={𝐐}_{2}\Lambda_{2}{𝐐}_{2}^{⊤}$.

3: **while** not converged **do**

4:  Update **W** by solving (15).

5:  Update **U** by solving (17).

6:  Update **F** by solving (24).

7:  Update **M** by solving (26).

8:  Update **P**, *μ*, **Z** and *σ* according to (27)

**end while**

**return W** and **M**.

### Prediction

Once the optimal **W** and **M** are obtained, the label l(x) for a given test instance x will be given by


$$l(x)=\arg\min_{k}\left({𝐖}^{⊤}x-y^{k}{)}^{⊤}𝐌({𝐖}^{⊤}x-y^{k}\right)$$ (28)

where $y^{k}$ is the regression target encoding specified for the *k*th class, k=1,…,c.

### Complexity and convergence analysis

As seen in Algorithm 1, the main time cost is solving Sylvester equations and computing **M**. The Schur decompositions of ${𝐗}^{⊤}𝐋𝐗$ and ${𝐗}^{⊤}𝐃𝐗$ are performed only once outside the loop, with computational complexity *O*(*d*^3^). The computational complexity of **M** and ${𝐌}^{-1}$ in each iteration is *O*(*r*^3^). For **F**, the computational complexity is *O*(*nc*). The computations of Lagrange multiplier **P**, **Z** and penalty factor *μ*, *σ* are very simple, and thus their computational costs can be ignored. Assuming *t* is the number of iterations, we can conclude that the total computational complexity of RMLCC is abou*t* $O(d^{3}+t(r^{3}+nc))$.

The optimization problem (13) is non-convex with respect to all unknown variables, it is difficult to prove the strong convergence property [13] of the algorithm. It is worth noting that Karush-Kuhn-Tucker (KKT) conditions are the necessary conditions for a constrained local optimal solution, and any converging point must be a KKT point. The following theorem guarantees a weak convergence property of the proposed optimization algorithm.

**Theorem 1.** *Let $\Theta^{k}≜({𝐖}^{k},{𝐔}^{k},{𝐅}^{k},{𝐌}^{k},{𝐏}^{k},{𝐙}^{k})$ be the solution of (13) at the kth iteration. Assume the sequence $\{\Theta^{k}{\}}_{k=1}^{\infty }$ is bounded and $\lim_{k\to \infty }(\Theta^{k+1}-\Theta^{k})=0$, then every limit point of $\{\Theta^{k}{\}}_{k=1}^{\infty }$ is a Karush-Kuhn-Tucker (KKT) point of the problem (13). Whenever $\{\Theta^{k}{\}}_{k=1}^{\infty }$ converges, it converges to a KKT point.*

*Proof.* Please refer to the Appendix S2 Appendix for the detailed proof of Theorem 1. □

### Experiment

In this section, we conduct several experiments to prove the effectiveness of the proposed method on six benchmark datasets. The main information of the six datasets is listed in Table 1. In particular, the proposed method was compared to some related state-of-the-art methods: DLSR [2], ReLSR [7], MSDLSR [6], RLSL [10], ICS_DLSR [8], GLRRDLR [9], PRDR [11]. For each group of experiments, all methods are repeated 10 times with the random combinations of training and test instances. All experiments are implemented on MATLAB R2017a with Win7 system, Inter Core i7-8550 CPU and 8GB RAM.

**Table 1:** Brief description of the benchmark datasets used in experiments.

Data set	# Classes	# Instances	# Features
Extended Yale B	38	2414	1024
AR	120	3120	1024
LFW	86	1251	1024
PIE	68	11554	1024
COIL100	100	7200	1024
Fifteen Scene	15	4485	3000

### Experiments on face databases

In this part, four challenging face databases are chosen to evaluate these methods. All of the facial images have been cropped and resized to 32 × 32 pixels.

(1) **Extended Yale B** [17]: This dataset contains face images of 38 individuals, totaling 2414 facial images. We randomly select 15, 20, 25 and 30 images from each class as the training set and use the rest for testing. The results are listed in Table 2. From the experimental results, ICS_DLSR and GLRRDLR perform better than DLSR and ReLSR. PRDR achieves better performance than ICS_DLSR and GLRRDLR. RMLCC achieves the best performance by taking into account both inter-class margin and intra-class consistency.
(2) **AR** [18]: The database contains over 4000 face images of 126 individuals. We select a subset containing 3120 grey scale images from 120 subjects. For each individual, we randomly select 4, 6, 8, and 12 images for training, and the rest for testing. The experimental results are reported in Table 3. It should be noted that the classification accuracy of ICS DLSR and GLRRDLR are about 5% higher than that of DLSR, ReLSR, MSDLSR and RLSL when the number of training instances is small, because they consider the inter-class margin and intra-class similarity. PRDR exploits both the local relationship information from the instance space and the label space, achieving the performance comparable to RMLCC. RMLCC achieves the best performance by performing metric learning and group consistency to effectively capture the structural information in data.
(3) **LFW** [19]: This is a challenging large wild image dataset. Here, we use a subset consisting of 1251 images from 86 individuals with only 10–20 images per subject. We randomly select 5, 6, 7, 8 images of each subject as training instances. The remaining face images are used as test instances. Table 4 reports the experimental results on this dataset. It is obvious that the classification accuracies of all methods are relatively low, proving that the LFW database is a very challenging database for face recognition. The accuracies of DLSR, ReLSR, MSDLSR and RLSL are grouped together at the lowest level. Encouragingly, the proposed RMLCC still came top. GLRR and PRDR are comparable to RMLCC, and more robust than DLSR and ReLSR. These experimental results prove the superiority of the proposed method on challenging data sets.
(4) **CMU PIE** [20]: This dataset contains 68 person with 41368 face images in total. Here, we compare all methods on a subset of PIE, where each person has 170 images, gathered under five different poses (C05, C07, C09, C27 and C29). As can be seen from Table 5, the proposed method is superior to the other regression methods on the basis of label relaxation. For example, the classification accuracy of the proposed method is up to about 4% higher than that of DLSR. RMLCC uses metric learning and class-specific consistency constraint to explore the inter-class margin and intra-class similarity. As a result, its classification accuracy is higher than that of PRDR.

**Table 2:** Mean classification accuracies (%) and standard deviations of different methods on the Extended Yale B face database.

	# training instances per class			
Method	15	20	25	30
DLSR	92.13 ± 0.62	93.64 ± 0.75	95.68 ± 0.63	96.39 ± 0.53
ReLSR	93.04 ± 0.73	94.25 ± 0.46	96.23 ± 0.42	97.21 ± 0.46
MSDLSR	93.37 ± 0.81	94.78 ± 0.77	96.71 ± 0.73	97.55 ± 0.69
RLSL	93.68 ± 0.80	95.10 ± 0.60	97.12 ± 0.53	97.89 ± 0.61
ICS_DLSR	93.89 ± 0.58	96.30 ± 0.63	97.36 ± 0.46	98.01 ± 0.52
GLRRDLR	94.07 ± 0.58	96.55 ± 0.68	97.69 ± 0.38	98.36 ± 0.43
PRDR	94.27 ± 0.71	96.76 ± 0.70	97.84 ± 0.45	98.59 ± 0.42
RMLCC	**94.61 ± 0.57**	**96.93 ± 0.66**	**98.23 ± 0.63**	**99.41 ± 0.37**

**Table 3:** Mean classification accuracies (%) and standard deviations of different methods on the AR face database.

	# training instances per class			
Method	4	6	8	12
DLSR	86.33 ± 0.73	90.83 ± 0.51	93.88 ± 0.49	96.57 ± 0.46
ReLSR	87.12 ± 0.77	91.97 ± 0.69	95.43 ± 0.55	97.34 ± 0.51
MSDLSR	87.89 ± 0.71	92.38 ± 0.77	95.13 ± 0.73	97.76 ± 0.69
RLSL	88.23 ± 0.74	92.66 ± 0.68	95.88 ± 0.66	98.02 ± 0.63
ICS_DLSR	91.27 ± 0.57	95.66 ± 0.47	97.41 ± 0.42	98.52 ± 0.41
GLRRDLR	91.73 ± 0.66	95.93 ± 0.58	97.77 ± 0.49	98.64 ± 0.56
PRDR	92.22 ± 0.73	95.66 ± 0.71	97.64 ± 0.55	98.73 ± 0.53
RMLCC	**92.51 ± 0.77**	**96.58 ± 0.66**	**98.87 ± 0.33**	**99.33 ± 0.51**

**Table 4:** Mean classification accuracies (%) and standard deviations of different methods on the LFW face database.

	# training instances per class			
Method	5	6	7	8
DLSR	30.38 ± 1.06	32.28 ± 0.91	34.22 ± 0.88	36.25 ± 0.76
ReLSR	31.68 ± 1.11	34.33 ± 1.08	37.48 ± 0.98	39.19 ± 0.87
MSDLSR	32.23 ± 1.07	34.98 ± 1.01	37.88 ± 1.05	39.39 ± 0.88
RLSL	35.81 ± 0.99	38.43 ± 0.89	40.92 ± 0.98	42.38 ± 0.82
ICS_DLSR	37.32 ± 1.03	39.77 ± 0.97	41.88 ± 0.76	42.92 ± 0.68
GLRRDLR	38.16 ± 0.97	42.87 ± 0.87	43.62 ± 0.75	44.61 ± 0.70
PRDR	38.31 ± 1.03	43.10 ± 0.71	43.74 ± 0.81	44.83 ± 0.73
RMLCC	**38.70 ± 0.77**	**43.79 ± 0.66**	**44.13 ± 0.68**	**45.43 ± 0.61**

**Table 5:** Mean classification accuracies (%) and standard deviations of different methods on the PIE face database.

	# training instances per class			
Method	15	20	25	30
DLSR	90.38 ± 0.85	91.39 ± 0.79	93.50 ± 0.75	93.85 ± 0.66
ReLSR	91.15 ± 0.82	92.43 ± 0.77	93.60 ± 0.71	94.35 ± 0.63
MSDLSR	91.18 ± 0.91	92.56 ± 0.88	93.82 ± 0.79	94.89 ± 0.68
RLSL	91.31 ± 0.87	92.93 ± 0.74	93.72 ± 0.78	94.68 ± 0.59
ICS_DLSR	91.43 ± 0.86	93.26 ± 0.78	94.21 ± 0.74	95.38 ± 0.64
GLRRDLR	91.64 ± 0.91	93.57 ± 0.87	94.32 ± 0.55	96.67 ± 0.46
PRDR	92.43 ± 0.66	93.78 ± 0.49	94.87 ± 0.41	97.13 ± 0.33
RMLCC	**92.83 ± 0.73**	**93.87 ± 0.66**	**95.52 ± 0.63**	**97.66 ± 0.41**

The experimental results presented in Tables 2, Table 3, Table 4, Table 5 show that the proposed method achieves the best classification accuracy in comparison with the related methods, which indicates its effectiveness in face recognition.

### Experiments on the object database

In this subsection, the Columbian Object Image Library (COIL 100) [21] is chosen for the evaluation of the effectiveness of the proposed method. This database consists of 7200 images of 100 objects. Each object has 72 images in different lighting conditions. For each class, we randomly select 15, 20, 25, and 30 images for training and the rest for testing. As can be seen from the results in Table 6, our method outperforms all of the comparison methods.

**Table 6:** Mean classification accuracies (%) and standard deviations of different methods on the COIL100 database.

	# training instances per class			
Method	15	20	25	30
DLSR	88.14 ± 0.79	90.21 ± 0.51	92.21 ± 0.55	93.43 ± 0.67
ReLSR	89.26 ± 0.73	90.33 ± 0.66	92.36 ± 0.53	93.55 ± 0.71
MSDLSR	90.18 ± 0.77	91.66 ± 0.61	92.88 ± 0.56	94.56 ± 0.68
RLSL	90.88 ± 0.71	93.13 ± 0.74	93.91 ± 0.55	94.36 ± 0.51
ICS_DLSR	91.33 ± 0.66	92.88 ± 0.55	93.88 ± 0.49	94.87 ± 0.51
GLRRDLR	91.21 ± 0.67	94.20 ± 0.40	95.47 ± 0.44	96.54 ± 0.42
PRDR	92.48 ± 0.53	94.51 ± 0.42	95.57 ± 0.37	96.93 ± 0.33
RMLCC	**92.91 ± 0.61**	**95.43 ± 0.60**	**96.33 ± 0.55**	**97.36 ± 0.40**

**Fig 2:**
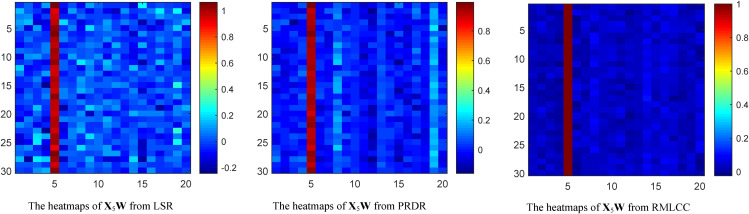
Class-specific consistency heatmaps of the transformation matrix obtained by LSR, PRDR and RMLCC.

**Fig 3:**
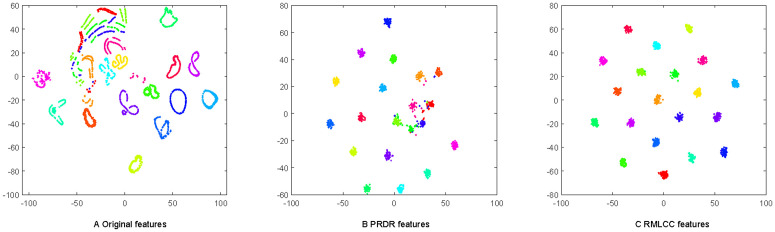
t-SNE visualization of original data, PRDR and RMLCC features on COIL100 dataset. The 1440 instances from the first 20 classes are visualized when 30 images per subject are used for training. Both training instances and testing instances are visualized.

As discussed in Section 5, the class-specific consistency (i.e., only a single nonzero column of **X**_*j*_**W**) is essential to ensure the intra-class similarity of the instances. To demonstrate the preservation of intra-class similarity by our method, Fig 2 shows the transformation matrix **X**_5_**W** of LSR, PRDR and RMLCC on the instances from the top 20 class of the COIL100 dataset. One can see that, the largest elements of the transformed instances **X**_5_**W** obtained by our method are all located in the fifth column, and the other columns are almost 0. This ensures the intra-class consistency and can effectively avoid overfitting. To further visually demonstrate the discriminative ability of the RMLCC, Fig 3 shows the t-SNE [22]visualization of the original instances and transformed features learned by RMLCC. For clarity, we selected 1440 instances from the top 20 classes for visualisation. It is clear from Fig 3A that the instance distributions of some classes are highly scattered and even overlap. Fig 3B shows better clustering by class than Fig 3A. However, in the right half of Fig 3B, the data distributions of the different classes still overlap. As shown in Fig 3C, our RMLCC can ensure that transformed features of the same class lie close together, while features of different classes lie further apart. This confirms the validity of the proposed method.

### Experiments on the scene database

Here, we use the spatial pyramidal features of the Fifteen Scene Categories database (Scene15_SPM) [23] to evaluate the proposed method. This data set contains 4485 natural images from 15 different classes, such as bedroom, industry, coast, street and building. We randomly select 10, 20, 30 and 40 instances from each class as the training set, and the remaining as the test set. The experimental results on the Scene15_SPM dataset are reported in Table 7. From Table 7, we can see that our method achieves the best performance among all the methods. This demonstrates the effectiveness of our method in dealing with the scene classification task. Additionally, Fig 4 shows the confusion matrix of our RMLCC on the Scene15_SPM dataset. Specifically, the classification accuracy (%) for the corresponding class is given by the diagonal elements of the confusion matrix. Notably, all classes achieved high classification accuracies, and the worst performance is still acceptable at 95.64%, also reflecting the superiority of our RMLCC.

**Table 7:** Mean classification accuracies (%) and standard deviations of different methods on the Scene15_SPM database.

	# training instances per class			
Method	10	20	30	40
DLSR	84.46 ± 0.99	89.53 ± 0.87	89.88 ± 0.77	91.12 ± 0.72
ReLSR	87.69 ± 0.97	91.51 ± 0.88	93.47 ± 0.71	94.33 ± 0.68
MSDLSR	88.92 ± 0.83	92.67 ± 0.77	94.51 ± 0.71	95.56 ± 0.63
RLSL	87.69 ± 0.91	92.33 ± 0.74	94.91 ± 0.73	96.83 ± 0.69
ICS_DLSR	89.73 ± 0.95	93.34 ± 0.77	95.68 ± 0.71	95.87 ± 0.51
GLRRDLR	89.75 ± 0.92	93.44 ± 0.66	95.37 ± 0.61	96.93 ± 0.57
PRDR	89.10 ± 0.97	93.28 ± 0.77	95.78 ± 0.66	96.79 ± 0.39
RMLCC	**90.73 ± 0.92**	**94.51 ± 0.85**	**96.33 ± 0.83**	**97.36 ± 0.76**

**Fig 4:**
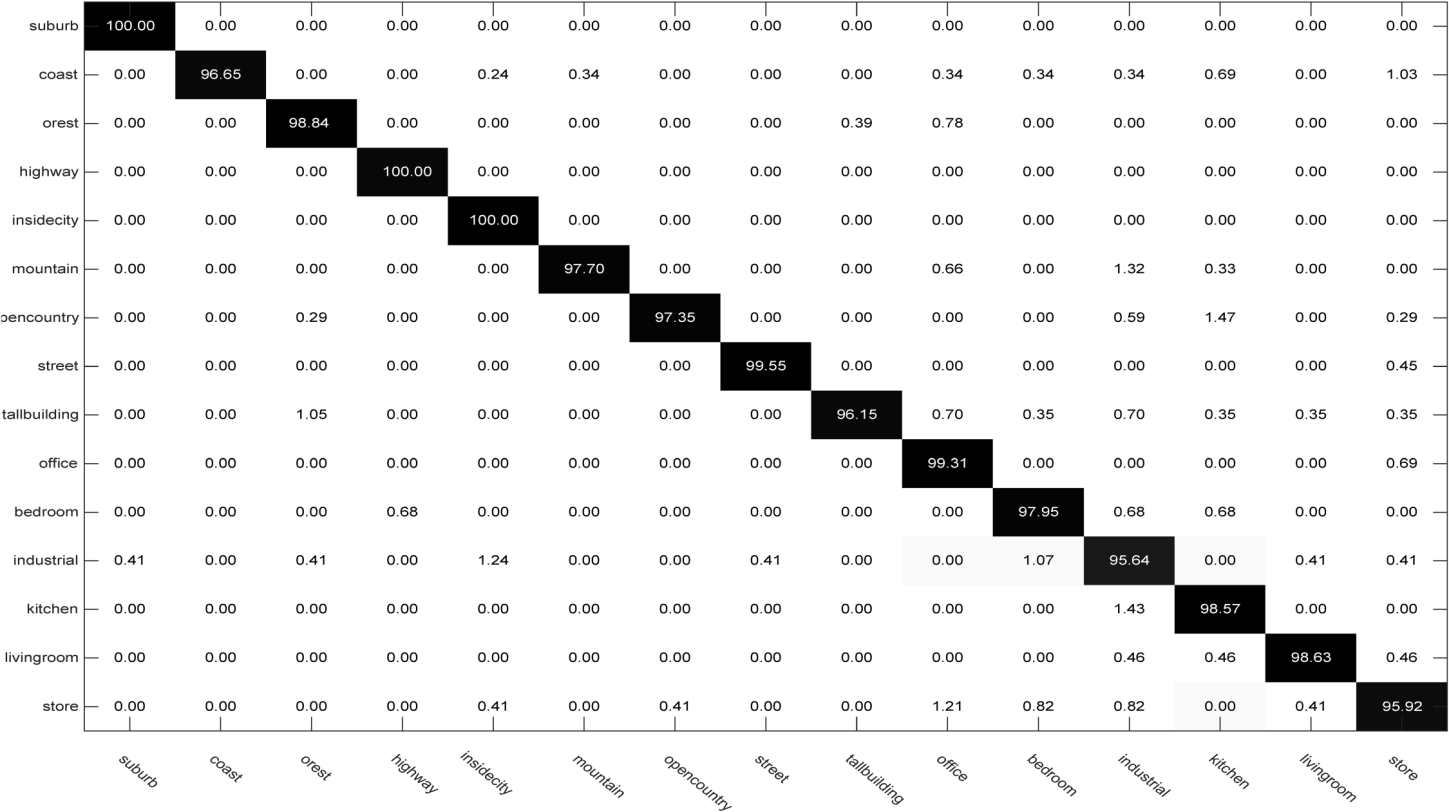
Confusion matrices of RMLCC on the Scene15_SPM dataset.

### Statistical significance

**Fig 5:**
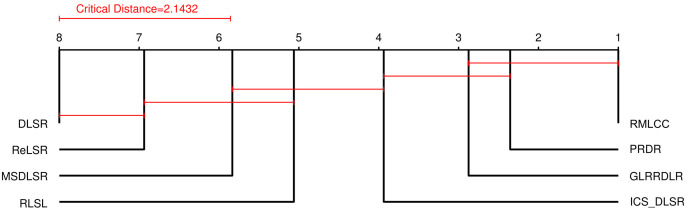
CD diagram of different methods with significance level α=0.05.

To systematically compare the different methods, the Friedman test and post hoc Nemenyi test [24] are used to compare the classification accuracy of the eight methods on six benchmark datasets with different numbers of training instances. In the experiments, two algorithms are regarded as significantly different if their average ranks differ by at least the Critical Difference (CD). Fig 5 shows the CD diagrams for the eight comparison methods on the six benchmark datasets with different numbers of training instances. The average rank of each comparison method is marked along the axis. The axis is turned so that the lowest ranks (best performance) are on the right. The methods in the groups linked by a red line are not significantly different. As shown in Fig 5, the ICS_DLSR, RLSL, MSDLSR, ReLSR and DLSR rank highly and are significantly different from the RMLCC. This result shows that RMLCC is well ahead of most other methods.

### Convergence and computational performance

**Fig 6:**
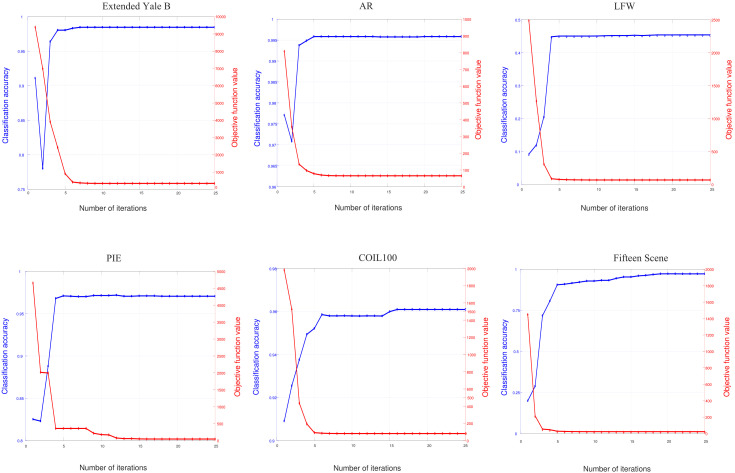
Convergence curve and classification accuracy versus iterations.

Here we experimentally demonstrate the good convergence of RMLCC. Fig 6 shows the variation curve of the classification accuracy with respect to the number of iterations and the convergence curve, where the blue line is the classification accuracy curve and the red line is the convergence curve. The classification accuracy increases rapidly in the previous iterations, while remaining stable after 15 iterations. In addition, the objective value decreases in a monotonic way with the number of iterations. The above results demonstrate the effectiveness of the proposed optimization method.

To ascertain the computational complexity of the proposed method, Table 8 reports the training time of the compared methods. PRDR has the shortest training time, while RLSL has the longest training time on most datasets. Our RMLCC is relatively slow compared to PRDR, DLSR, and MSDLSR, ranking third in terms of training speed. The main reason is that RMLCC needs to solve Sylvester equations in each iteration. Although the execution time for the RMLCC is slightly longer, it is possible to use off-line training for tasks that require a higher prediction accuracy.

**Table 8:** Run time (second) comparisons of different methods.

	Data					
Method	Extended Yale B	AR	LFW	PIE	COIL100	Scene15_SPM
	30×38	12×120	8×86	30×68	30×100	40×15
DLSR	4.72	7.31	3.09	6.50	14.54	10.93
ReLSR	14.04	36.89	13.87	32.17	67.45	44.76
MSDLSR	8.68	10.63	6.24	13.11	21.54	36.03
RLSL	30.12	61.85	29.65	57.18	112.11	97.11
ICS_DLSR	10.46	13.44	8.06	14.38	37.53	84.27
GLRRDLR	15.33	32.03	21.28	24.44	62.76	50.76
PRDR	2.95	5.21	2.43	4.15	19.55	7.22
RMLCC	8.71	10.88	7.33	14.21	28.76	37.53

### Parameter sensitivity and setting

**Fig 7:**
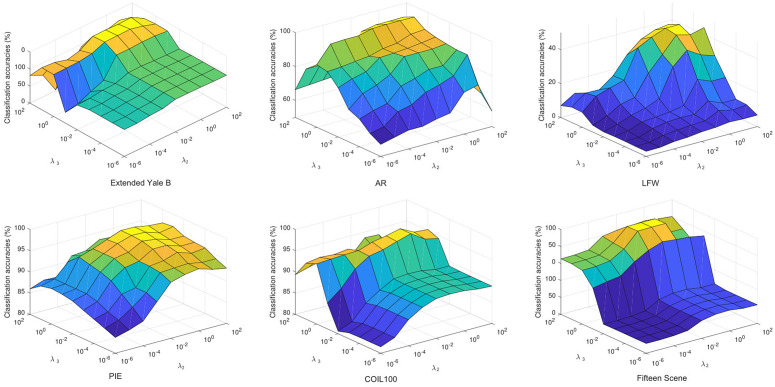
The change in classification accuracy with the $\lambda_{2}$ and $\lambda_{3}$ on the datasets.

In RMLCC, there are three parameters $\lambda_{1}$, $\lambda_{2}$ and $\lambda_{3}$, which serve to balance the significance of each item in the object. To analyze the parameter sensitivity of RMLCC, a candidate set $\left\{10^{-6},10^{-5},10^{-4},10^{-3},10^{-2},10^{-1},10^{0},10^{1},10^{2}\right\}$ was first defined for $\lambda_{1}$, $\lambda_{2}$ and $\lambda_{3}$. The study revealed that the model’s performance is not sensitive to the $\lambda_{1}$, and the performance remains stable when $\lambda_{1}<10^{-4}$. Therefore, we fix $\lambda_{1}$ at the optimal value of $10^{-6}$ and analysis the effect of $\lambda_{2}$ and $\lambda_{3}$ on the performance of the model. Fig 7 shows the variation of the classification performance of our method with respect to different values of $\lambda_{2}$ and $\lambda_{3}$. Obviously, the proposed method can achieve satisfactory performance when $\lambda_{1}$ and $\lambda_{2}$ are located in $\left[10^{-2},1\right]$ and $\left[10^{-2},10^{1}\right]$, respectively. In addition, a geometric mean weight *α* needs to be tuned in the model, while it is actually close to 0 in most cases. We select it from the set $\left\{10^{-6},10^{-5},10^{-4},10^{-3},10^{-2}\right\}$ in the experiments. The prior matrix **M**_0_ was set to the identity matrix throughout the experiment.

## Conclusion

In this paper, a novel method called residual metric learning with class-specific consistency (RMLCC) is proposed for multiclass classification. Different from existing methods, instead of directly fitting the original features to binary or relaxed labels, this proposed method jointly learns the instance transformation matrix and residual metric matrix in a unified framework, and imposes class-specific consistency constraints on the transformed instances. As a result, in the learned metric space, the distances between transformed instances of same class are as small as possible while the distances between different classes are as large as possible from each other, thus improving the discriminative power. Extensive experiments on the face, object, and scene databases validate the effectiveness of the proposed method.

In the future work, we intend to study the integration of higher-order structure of instances to further improve the discriminative expression ability of the model. In addition, the establishment of a non-linear metric learning method as a replacement for GMML to improve the discriminability for the complex structured data is also the focus of our future research.

## Supporting information

S1 Appendix Proof of the Proposition 1. (PDF)

S2 Appendix Proof of the Theorem 1. (PDF)
