# Supplementary material for: Residual metric learning with class-specific consistency for multiclass classification
Source: PLoS One. 2026 Mar 25;21(3):e0345369. doi: 10.1371/journal.pone.0345369 (PMC13016361; doi:10.1371/journal.pone.0345369)
Supplement: S1 Appendix — (PDF) [file pone.0345369.s001.pdf]

Residual metric learning with class-specific consistency for multiclass classification

Kai Hu [1], Jiajun Ma [1*]

1 School of Computer Science and Engineering, Xi'an Technological University, Xi'an, Shaanxi, China

* majiajun86@xatu.edu.cn

Supporting information

S1 Appendix. Proof of the Proposition 1.

Proof. To succinctly prove this proposition, the relevant norm definition is first introduced. Let $\boldsymbol{\alpha} = [a_1, a_2, \dots, a_c]$, where $a_j = \|\mathbf{A}_{:,j}\|_2 \geq 0$, and $\mathbf{A}_{:,j}$ denotes the jth column of $\mathbf{A}$. Then, we have $\|\mathbf{A}\|_{1,2} = \|\boldsymbol{\alpha}\|_1$, and $\|\mathbf{A}\|_{2,2} = \|\boldsymbol{\alpha}\|_2$. Thus, Proposition 1 is equivalent to: for any non-zero vector $\boldsymbol{\alpha}$, $\|\boldsymbol{\alpha}\|_1 - \|\boldsymbol{\alpha}\|_2 \geq 0$ and $\|\boldsymbol{\alpha}\|_1 - \|\boldsymbol{\alpha}\|_2 = 0$ if and only if $\boldsymbol{\alpha}$ has one single nonzero entry.

By the definition of the l_1-norm and the l_1-norm of the vectors, $\|\boldsymbol{\alpha}\|_1^2 = |a_1|^2 + |a_2|^2 + \ldots + |a_c|^2 + 2\Sigma_{i>j=1}^{c}|a_i||a_j| = \|\boldsymbol{\alpha}\|_2^2 + 2\Sigma_{i>j=1}^{c}|a_i||a_j| \geq \|\boldsymbol{\alpha}\|_2^2$. Thus, we have $\|\boldsymbol{\alpha}\|_1 - \|\boldsymbol{\alpha}\|_2 \geq 0$. It is obvious that $\|\boldsymbol{\alpha}\|_1^2 = \|\boldsymbol{\alpha}\|_2^2$ if and only if $\Sigma_{i>j=1}^{c}|a_i||a_j| = 0$. Given that all the terms in the sum $\Sigma_{i>j=1}^{c}|a_i||a_j|$ are nonnegative, the equation is hold if $\boldsymbol{\alpha} = 0$, or if $\boldsymbol{\alpha}$ has one single nonzero entry. That means $\|\mathbf{A}\|_{1,2} - \|\mathbf{A}\|_{2,2} \geq 0$ if either $\mathbf{A} = 0$ or $\mathbf{A}$ has one single nonzero column. □
