# Supplementary material for: Residual metric learning with class-specific consistency for multiclass classification
Source: PLoS One. 2026 Mar 25;21(3):e0345369. doi: 10.1371/journal.pone.0345369 (PMC13016361; doi:10.1371/journal.pone.0345369)
Supplement: S2 Appendix — (PDF) [file pone.0345369.s002.pdf]

Residual metric learning with class-specific consistency for multiclass classification

Kai Hu [1], Jiajun Ma [1*]

1 School of Computer Science and Engineering, Xi'an Technological University, Xi'an, Shaanxi, China

* majiajun86@xatu.edu.cn

Supporting information

S2 Appendix. Proof of the Theorem 1.

Proof. For simplicity, let $\mathcal{L}$ denotes the optimization problem (12). The KKT conditions for (12) are derived as follows (The process of solving $\mathbf{M}$ does not involve in the Lagrange multipliers, thus we do not proof the KKT condition for it):

$$\mathbf{U} = \mathbf{W}, \qquad \mathbf{F} = \mathbf{X}\mathbf{W}. \tag{S.1}$$

$$\mathbf{X}^\top \mathbf{L}(\mathbf{X}\mathbf{W} - \mathbf{Y})\mathbf{M} + \mathbf{P} + \mathbf{X}^\top \mathbf{Z} = 0. \tag{S.2}$$

$$\mathbf{X}^\top \mathbf{D}(\mathbf{X}\mathbf{U} - \mathbf{Y})\mathbf{M}^{-1} + \lambda_1 \mathbf{U} - \mathbf{P} = 0 \tag{S.3}$$

$$[\mathbf{Z}_j]_{:,k} + 2\lambda_3 [\mathbf{F}_j]_{:,k} \left(\frac{1}{\|[\mathbf{F}_j]_{:,k}\|_2} - \frac{1}{\|\mathbf{F}_j\|_{2,2}} \right) = 0 \tag{S.4}$$

First, the Lagrangian multiplier $\mathbf{P}$ and $\mathbf{Z}$ can be obtained from Algorithm 1, as follows

$$\mathbf{P}^k = \mathbf{P} + \mu(\mathbf{W} - \mathbf{U}), \mathbf{Z}^k = \mathbf{Z} + \sigma(\mathbf{X}\mathbf{W} - \mathbf{F}). \tag{S.5}$$

If sequence $\{\mathbf{P}^k\}_{k=1}^{\infty}$ and $\{\mathbf{Z}^k\}_{k=1}^{\infty}$ converge to the stationary points, i.e., $(\mathbf{P}^k - \mathbf{P}) \to 0$ and $(\mathbf{Z}^k - \mathbf{Z}) \to 0$, then $(\mathbf{U} - \mathbf{W}) \to 0$ and $(\mathbf{F} - \mathbf{X}\mathbf{W}) \to 0$. Thus, the first KKT condition (S.1) is proved.

The second KKT condition can also be derived by utilizing the result of $\mathbf{W}$ in Algorithm 1. We first rewrite (15) as follows:

$$\begin{aligned} \mu\mathbf{W} = &- \mathbf{X}^\top \mathbf{L}(\mathbf{X}\mathbf{W} - \mathbf{Y})\mathbf{M} - \mathbf{P} - \mathbf{X}^\top \mathbf{Z} \\ &+ \delta \mathbf{X}^\top (\mathbf{F} - \mathbf{X}\mathbf{W}) + \mu \mathbf{U} \end{aligned} \tag{S.6}$$

Then, we can obtain the following:

$$\begin{aligned} \mu(\mathbf{W}^k - \mathbf{W}) = &- \left(\mathbf{X}^\top \mathbf{L}(\mathbf{X}\mathbf{W} - \mathbf{Y})\mathbf{M} + \mathbf{P} + \mathbf{X}^\top \mathbf{Z}\right) \\ &+ \delta \mathbf{X}^\top (\mathbf{F} - \mathbf{X}\mathbf{W}) + \mu(\mathbf{U} - \mathbf{W}) \end{aligned} \tag{S.7}$$

If $(\mathbf{W}^k - \mathbf{W}) \to 0$, then $\mathbf{X}^\top \mathbf{L}(\mathbf{X}\mathbf{W} - \mathbf{Y})\mathbf{M} + \mathbf{P} + \mathbf{X}^\top \mathbf{Z} \to 0$.

Likewise, we can get the following equation using $\mathbf{U}$ from Algorithm 1:

$$\begin{aligned}&(\lambda_1+\mu)(\mathbf{U}^k-\mathbf{U})\\&=-\big(\mathbf{X}^\top\mathbf{D}(\mathbf{X}\mathbf{U}-\mathbf{Y})\mathbf{M}^{-1}+\lambda_1\mathbf{U}-\mathbf{P}\big)-\mu(\mathbf{U}-\mathbf{W})\end{aligned} \tag{S.8}$$

If $(\mathbf{U}^k-\mathbf{U})\to 0$, then $\mathbf{X}^\top\mathbf{D}(\mathbf{X}\mathbf{U}-\mathbf{Y})\mathbf{M}^{-1}+\lambda_1\mathbf{U}-\mathbf{P}\to 0$.

For the fourth condition, from (23), we have the following equation:

$$\begin{aligned}&[\mathbf{F}_j]_{l,k}^{t+1}-[\mathbf{F}_j]_{l,k}^{t}\\&=\frac{\sigma^t[\mathbf{H}_j]_{l,k}}{\sigma^t+\lambda_3(\frac{1}{\|[\mathbf{F}_j]^t\|_{2,2}}-\frac{1}{\|[\mathbf{F}_j]_{:,k}^t\|_2})}-[\mathbf{F}_j]_{l,k}^t\end{aligned} \tag{S.9}$$

As $\mathbf{H}=\mathbf{X}\mathbf{W}+\frac{\mathbf{Z}}{\sigma}$, if $\{\mathbf{F}^t\}_{t=1}^\infty$ converges, then we have

$$\begin{aligned}&[\mathbf{F}_j]_{:,k}^{t+1}-[\mathbf{F}_j]_{:,k}^{t}\\&=\frac{\sigma^t\boldsymbol{h}_k}{\sigma^t+2\lambda_3(\frac{1}{\|\boldsymbol{f}_k^t\|_2}-\frac{1}{(\sum_{k=1}^c\|\boldsymbol{f}_k^t\|_2^2)^{1/2}})}-\boldsymbol{f}_k^t\\&=\frac{\sigma^t([\mathbf{X}_j\mathbf{W}^{t+1}]_{:,k}-[\mathbf{Z}_j^t/\sigma^t]_{:,k})}{\sigma^t+2\lambda_3(\frac{1}{\|\boldsymbol{f}_k^t\|_2}-\frac{1}{(\sum_{k=1}^c\|\boldsymbol{f}_k^t\|_2^2)^{1/2}})}-\boldsymbol{f}_k^t\\&=\frac{\sigma^t([\mathbf{X}_j\mathbf{W}^{t+1}]_{:,k}-\boldsymbol{f}_k^t)}{\sigma^t+2\lambda_3(\frac{1}{\|\boldsymbol{f}_k^t\|_2}-\frac{1}{(\sum_{k=1}^c\|\boldsymbol{f}_k^t\|_2^2)^{1/2}})}\\&\quad-\frac{[\mathbf{Z}_j^t]_{:,k}+2\lambda_3\boldsymbol{f}_k^t(\frac{1}{\|\boldsymbol{f}_k^t\|_2}-\frac{1}{(\sum_{k=1}^c\|\boldsymbol{f}_k^t\|_2^2)^{1/2}})}{\sigma^t+2\lambda_3(\frac{1}{\|\boldsymbol{f}_k^t\|_2}-\frac{1}{(\sum_{k=1}^c\|\boldsymbol{f}_k^t\|_2^2)^{1/2}})}\end{aligned} \tag{S.10}$$

If $([\mathbf{F}_j]_{:,k}^{t+1}-[\mathbf{F}_j]_{:,k}^{t})\to 0$, Utilizing Eq. (S.1) and let $t\to\infty$, then $[\mathbf{Z}_j^t]_{:,k}+2\lambda_3\boldsymbol{f}_k^t(\frac{1}{\|\boldsymbol{f}_k^t\|_2}-\frac{1}{(\sum_{k=1}^c\|\boldsymbol{f}_k^t\|_2^2)^{1/2}})\to 0$. According to the definition of $\boldsymbol{f}_k$, we obtain $[\mathbf{Z}_j]_{:,k}+2\lambda_3[\mathbf{F}_j]_{:,k}$
$\Big(\frac{1}{\|[\mathbf{F}_j]_{:,k}\|_2}-\frac{1}{\|\mathbf{F}_j\|_{2,2}}\Big)\to 0$.

Since $\{\Theta^k\}_{k=1}^\infty$ is bound by the assumption in Theorem 1. As a result, $\lim\limits_{k\to\infty}(\Theta^{k+1}-\Theta^k)=0$ can deduce that both sides of (S.2), (S.3) and (S.4)are approximate to 0 when $k\to\infty$. Thus, the value of sequence $\{\Theta^k\}_{k=1}^\infty$ asymptotically satisfies the KKT condition for objective function (12). □
